# A randomized, controlled, repeat-dose study of batefenterol/fluticasone furoate compared with placebo in the treatment of COPD

**DOI:** 10.1186/s12890-020-1153-7

**Published:** 2020-05-04

**Authors:** Courtney Crim, Mark Gotfried, Selwyn Spangenthal, Michael Watkins, Amanda Emmett, Catriona Crawford, Charlotte Baidoo, Ramiro Castro-Santamaria

**Affiliations:** 1 GSK, Research & Development, Research Triangle Park, NC USA; 2 Pulmonary Associates, Phoenix, AZ USA; 3 American Health Research, Charlotte, NC USA; 4 PAREXEL International, Durham, NC USA; 5 GSK, Global Medical, Stockley Park West, 1–3 Ironbridge Road, Uxbridge, Middlesex UK; 6 GSK, Clinical Statistics, Stockley Park West, 1–3 Ironbridge Road, Uxbridge, Middlesex UK; 7 GSK, Research and Development, Collegeville, PA USA

**Keywords:** Bronchodilator, Bi-functional molecule, Triple therapy, Safety

## Abstract

**Background:**

Batefenterol (BAT) is a bi-functional molecule with both muscarinic antagonist and β_2_-adrenoceptor agonist pharmacology. This Phase II, randomized, placebo-controlled, double-blind study evaluated the safety and tolerability of BAT 300 μg with fluticasone furoate (FF) 100 μg administered via the ELLIPTA inhaler (BAT/FF 300/100).

**Methods:**

Subjects with stable chronic obstructive pulmonary disease were randomized 2:1 to receive BAT/FF 300/100 or placebo once daily for 6 weeks. The primary endpoint was change from baseline in 0–4-h weighted mean (WM) heart rate (HR, measured by electrocardiogram [ECG]) on Day 42. Other endpoints included WM and maximum 0–4-h corrected QT interval (ECG on Days 1, 28, and 42), HR measured by Holter monitoring (Day 42), and standard safety assessments. Study protocol was approved by an Investigational Review Board.

**Results:**

Sixty-two patients were randomized and received ≥1 dose of study medication (BAT/FF 300/100 *n* = 42; placebo *n* = 20). Mean age was 62.5 years (standard deviation [SD] 8.17). Study completion rates were 83% (BAT/FF 300/100) and 100% (placebo). Screening mean (SD) post-bronchodilator percentage-predicted forced expiratory volume in 1 s was 57.57 (11.42) in the BAT/FF 300/100 group and 55.68 (14.03) in the placebo group. BAT/FF 300/100 was non-inferior to placebo for the primary endpoint, treatment difference: − 2.2 beats per minute (bpm), 95% confidence interval [CI]: − 6.2, 1.7). There were no clinically relevant differences between treatment groups in WM or maximum 0–4-h corrected QT interval, or mean HR based on Holter monitoring on Day 42 (BAT/FF 300/100: 76.3 bpm [SD 11.38]; placebo: 84.8 bpm [SD 9.87]). Adverse events (AEs) occurred in 38% (BAT/FF 300/100) and 35% (placebo) of patients. AEs in ≥2 subjects with BAT/FF 300/100 were dysgeusia (10%), diarrhea (7%), nasopharyngitis (7%), and cough (5%). AEs leading to discontinuation occurred in two subjects who received BAT/FF 300/100: post-treatment severe pneumonia (serious AE) and non-serious AEs of moderate vomiting and severe gastroenteritis; both were not considered drug-related. No deaths occurred.

**Conclusions:**

Six weeks of BAT/FF 300/100 treatment was non-inferior to placebo for change from baseline in HR, with no new clinically relevant general or cardiovascular safety signals.

**Trial registration:**

Clinicaltrials.gov: NCT02573870 (submitted October 12, 2015).

## Background

Chronic obstructive pulmonary disease (COPD) is characterized by airflow obstruction and reduced maximum expiratory flow from the lungs that is not fully reversible [1, 2]. Inhaled bronchodilator therapy with long-acting β_2_-adrenergic agonists (LABAs) and long-acting muscarinic antagonists (LAMAs) are recommended for the maintenance treatment of most patients with COPD [1, 2]. Improved bronchodilation is achieved with combined LABA/LAMA treatment, compared with either monotherapy [1–3].

The addition of an inhaled corticosteroid (ICS) to LABA therapy is another strategy to improve outcomes in patients with COPD, particularly in those who continue to experience exacerbations despite bronchodilator therapy [3]. In recent years, there has been increasing interest in triple therapy (LABA/LAMA/ICS) to provide further improvements for patients with COPD who are not adequately controlled with dual combination therapy [4, 5]. Fluticasone furoate (FF) is a potent once-daily ICS, currently available in combination with the LABA vilanterol (VI) as a treatment for patients with COPD in the United States (US) and Europe, and as triple therapy with VI and umeclidinium, a LAMA, as maintenance treatment for patients with COPD in the US.

Batefenterol (BAT) is a novel bi-functional molecule with both muscarinic (M_2_ and M_3_ receptor) antagonist and β_2_-adrenoceptor agonist pharmacology (muscarinic antagonist, β_2_ agonist [MABA]) in development for the treatment of COPD [6, 7]. As well as simplifying the formulation for triple therapies [7, 8], using a MABA in combination with an ICS has the potential to improve efficacy through the delivery of a fixed ratio of LABA and LAMA to every region of the lung [3].

Early studies suggested that the use of LABAs was associated with an increased HR, reduced potassium concentration, and an increased risk of adverse cardiovascular (CV) events including ventricular tachycardia, acute coronary syndrome (myocardial infarction or unstable angina), congestive heart failure, and sudden cardiac death [9–11]. LAMA use has also been associated with an increased risk of adverse CV events [9]. This may be of particular relevance, given that individuals with COPD are already at increased risk of CV events compared with the general population [12].

This study is the first in which BAT 300 μg combined with FF 100 μg in a single inhaler (BAT/FF 300/100) was administered to patients with COPD. This study was undertaken to evaluate the safety and tolerability, particularly the CV safety, of BAT/FF 300/100 administered once daily compared with placebo in subjects with COPD. Additionally, the efficacy of BAT/FF 300/100 compared with placebo was explored, including their effects on patient-reported outcomes (PROs), as were the relationships between BAT pharmacokinetics (PK) and the pharmacodynamics (PD) of BAT/FF 300/100.

## Methods

This was a Phase IIa, multicenter, randomized, placebo-controlled, double-blind, parallel-group study (GSK study number 201546; www.clinicaltrials.gov registration number NCT02573870) undertaken between December 9, 2015 and July 5, 2016, in 10 US centers. The study protocol and informed consent forms were reviewed and approved by national, regional, or investigational center ethics committees/institutional review boards in accordance with the International Conference on Harmonisation of Technical Requirements for Registration of Pharmaceuticals for Human Use Good Clinical Practice (GCP) guidelines.

The study was conducted in accordance with GCP and the ethical principles outlined in the Declaration of Helsinki (2008). All subjects provided written, informed consent prior to study participation.

### Subjects

Male and female subjects ≥40 years of age with an established clinical history of COPD, according to the American Thoracic Society/European Respiratory Society definition [1], were eligible for inclusion in the study if they had a post-albuterol forced expiratory volume in 1 s (FEV_1_)/forced vital capacity ratio ≤ 0.70 and a post-albuterol FEV_1_ ≥ 30 and ≤ 80% of predicted normal values [13]. Current or former cigarette smokers with ≥10 pack-years history at Screening (Visit 1) were eligible. Exclusion criteria included: poorly controlled COPD, defined as ‘acute worsening of COPD that is managed with systemic corticosteroids and/or antibiotics or that requires treatment prescribed by a physician in the 6 weeks prior to Screening’, or hospitalization due to acute worsening of COPD within 12 weeks of Screening; more than one COPD exacerbation (moderate or severe) within the 12 months prior to Screening; other respiratory disorders (a current diagnosis of asthma; known α-1 antitrypsin deficiency; active lung infections such as tuberculosis, pneumonia or lower respiratory tract infection; or lung cancer); or had oxygen therapy prescribed for more than 12 h per day. Further details regarding the inclusion and exclusion criteria are provided in Additional file 1: Supplementary Appendix. Use of excluded medications (Table S1) was not permitted within the defined time periods prior to Screening and throughout the study.

Randomization exclusion criteria included: COPD exacerbation/lower respiratory infection requiring treatment other than albuterol recue medication during the run-in period; abnormal clinically significant laboratory findings in any liver chemistry, biochemical, or hematology tests; abnormal and clinically significant 12-lead electrocardiogram (ECG); or an abnormal and significant finding from 24-h Holter monitoring.

### Study design

Subjects who met the inclusion criteria and none of the exclusion criteria at Screening entered a 7-day run-in period. Subjects who did not meet any of the randomization exclusion criteria at the end of the run-in period were randomized to receive study treatment for a 6-week treatment period with clinic visits at Week 1, 2, 4, and 6. A follow-up visit occurred 1 week after the treatment period ended. Inhaled albuterol was provided from Screening to the end of the treatment period for all subjects to use as needed to relieve COPD symptoms.

### Treatment

**Fig. 1:**
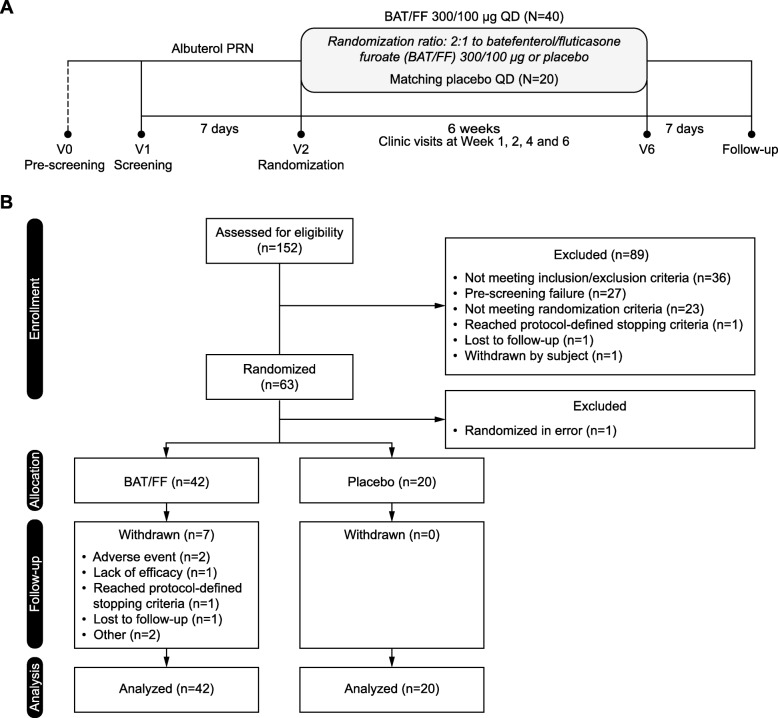
Study design (**a**) and participant flow diagram (**b**). BAT/FF, batefenterol/fluticasone furoate; PRN, Pro re nata (when necessary); QD, once daily; V, visit

Eligible subjects were randomized 2:1 during Visit 2 (Day 1) to receive BAT/FF 300/100 inhalation powder or placebo once daily for 6 weeks (Fig. 1a). Subjects were assigned to study treatments in accordance with the block randomization schedule generated by PAREXEL Informatics Inc. (Waltham, MA, USA), using validated internal software. This system used a computer-generated randomization schedule and an Interactive Voice Response System. The investigators, patients and the sponsor were blinded to the treatment assignment. Study treatments were self-administered, using a multi-dose dry powder inhaler (ELLIPTA-DPI), once daily in the morning for 42 days. Subjects were required to have compliance with study treatment ≥80% and ≤ 120% between each pair of on-treatment visits (scheduled or unscheduled) as assessed using the dose counter on the ELLIPTA. Subjects who fell outside this range were re-educated on treatment compliance.

### Endpoints

The primary endpoint was change from baseline (pre-dose on Day 1) in 0–4-h post-dose weighted mean (WM) heart rate (HR) (derived from 12-lead ECGs) at the end of the 6 weeks (42 days) of treatment. Additional CV safety endpoints included the WM and maximum 0–4 h Fridericia’s corrected QT interval [QTc(F)] derived from 12-lead ECGs recorded at several time points on Day 1, 28, and 42 during clinic visits, and Holter reading assessments on Day 42. Adverse events (AEs), incidence of COPD exacerbations (a worsening of COPD requiring treatment other than study medication or albuterol rescue medication), and vital sign measurements (pulse rate and systolic and diastolic blood pressure [BP]) were also captured.

Exploratory efficacy endpoints included change from baseline in 24-h trough FEV_1_ (derived from the mean of 2 pre-dose assessments taken 23 and 24 h after the previous dose of study treatment) on Day 7, 14, 28, and 42, and use of COPD rescue medication (albuterol) during a 24-h period averaged over the 6-week treatment period. The effect of BAT/FF 300/100 on PRO measures at Day 1 and Day 42 was evaluated using the COPD Assessment Test (CAT) and St George’s Respiratory Questionnaire (SGRQ).

PK endpoints included parameters derived from plasma concentrations of BAT on Day 1 and 42 collected pre-dose and 15 min, 30 min, 1 h, 2 h, and 4 h post dose. PD endpoints included WM fasting glucose and potassium concentrations, maximum fasting glucose concentrations, and minimum fasting potassium concentrations 0–4 h post-dose on Day 1 and 42. PK-PD relationships for BAT were also investigated.

### Statistical analysis

This non-inferiority study was designed to show that BAT/FF 300/100 was no worse than placebo for the primary endpoint. BAT/FF 300/100 would be declared non-inferior to placebo if the upper limit of the 2-sided 95% confidence interval (CI) for the estimated treatment difference for 0–4-h WM HR between the treatment groups was less than + 10 beats per minute (bpm), which was equivalent to a 1-sided hypothesis test at a 2.5% significance level. The non-inferiority margin of 10 bpm was established based on data from a previous COPD study with VI (NCT00372112), in which mean changes from baseline in the placebo group of up to 9.2 bpm were seen.

Based on a 1-sided hypothesis test at the 2.5% significance level and assuming a standard deviation for weighted mean heart rate of 10 bpm and a true difference of 0 bpm, 17 subjects in the placebo group and 34 in the BAT/FF 300/100 group would provide 90% power to demonstrate non-inferiority for the primary endpoint. It was planned to screen ~ 91 subjects to achieve a total of 60 subjects randomized to receive treatment (BAT/FF 300/100: 40; placebo: 20) and, assuming a 15% withdrawal rate, 51 subjects would complete the study.

All safety, exploratory efficacy, and PD analyses were conducted using data from the intent-to-treat (ITT) population, which comprised all randomized subjects who received at least one dose of study medication. The PK analysis was undertaken in the PK population, which comprised all subjects in the ITT population for whom a PK sample was obtained and analyzed.

For the primary endpoint, WM HR was derived by calculating the area under the curve (AUC) over 0–4 h post-dose and dividing by that length of time. The AUC was calculated using the trapezoid rule. Analysis of the primary endpoint was undertaken using mixed model repeated measures (MMRM) incorporating data from Day 1, 28, and 42, including the covariates baseline HR, gender, age, smoking status, nominal study day, treatment group, and treatment-group-by-day and baseline-by-day interactions. Baseline was defined as the pre-dose measurement on Day 1. As the primary objective of the study was to assess the safety and tolerability of BAT/FF 300/100, no multiplicity adjustments were made.

Analyses of all other safety and exploratory efficacy endpoints were undertaken using the same MMRM model as the primary endpoint, or an analysis of covariance model using the same covariates as for the primary endpoint. Data are presented as least squares (LS) mean change from baseline of the parameter of interest, with standard error for each treatment group and/or LS mean treatment differences with 95% CIs. PK-PD relationships were explored graphically using scatter plots.

## Results

### Subjects

In total, 62 subjects were randomized and received at least one dose of study medication (ITT population; Fig. 1b). Of these, 55 (89%) subjects completed the study; of the 7 (11%) subjects who withdrew, all were in the BAT/FF 300/100 group. A summary of subject demographics and baseline characteristics is shown in Table 1. Mean (standard deviation) age for the ITT population was 62.5 (8.17) years. Subject demographics and baseline characteristics were generally similar and balanced between the groups, with some exceptions; for example, there was a higher proportion of smokers at baseline in the placebo group compared with the BAT/FF 300/100 group (Table 1).

**Table 1:** Patient demographics and baseline characteristics

	BAT/FF 300/100 (***n*** = 42)	Placebo (***n*** = 20)
Age, years, mean (SD)	63.0 (7.88)	61.4 (8.86)
Range	45–78	45–77
Male, n (%)	18 (43)	8 (40)
BMI, kg/m^2^, mean (SD)	27.77 (5.44)	30.51 (8.43)
Range	18.7–43.0	20.4–50.2
Ethnicity, n (%)		
Hispanic or Latino	3 (7)	1 (5)
Not Hispanic or Latino	39 (93)	19 (95)
Race, n (%)		
American Indian or Alaskan Native	0	1 (5)
White – White/Caucasian/European heritage	36 (86)	16 (80)
White – Arabic/North African heritage	2 (5)	0
African American/African heritage	4 (10)	3 (15)
Current smoker, n (%)	21 (50)	16 (80)
Smoking pack-years, mean (SD)	51.3 (26.8)	41.0 (15.9)
Post-bronchodilator % predicted FEV_1_, mean (SD)^a^	57.57 (11.42)	55.68 (14.03)

^a^Spirometry data were obtained during screening

*BAT/FF 300/100* batefenterol/fluticasone furoate 300/100 μg, *BMI* body mass index, *FEV*_*1*_ forced expiratory volume in 1 s, *SD* standard deviation

### Safety

**Fig. 2:**
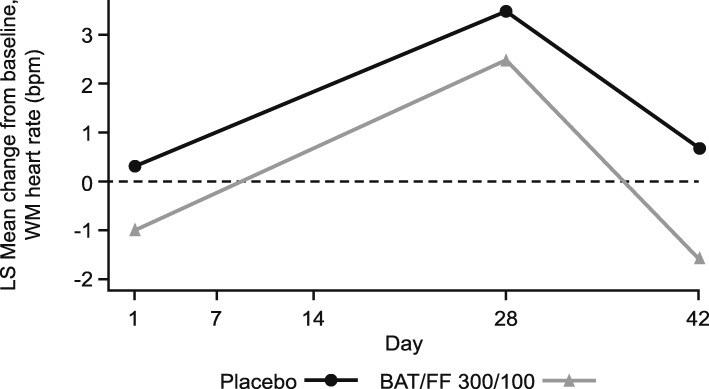
LS mean change from baseline in 0–4-h WM HR (bpm). BAT/FF 300/100, batefenterol/fluticasone furoate 300/100 μg; bpm, beats per minute; HR, heart rate; LS, least squares; WM, weighted mean

For the primary endpoint, the difference in LS mean change from baseline in 0─4-h WM HR on Day 42 in the BAT/FF 300/100 group compared with the placebo group was − 2.2 bpm (95% CI: − 6.2, 1.7) (Fig. 2; Additional file 1: Table S2). As the upper bound of the 95% CI was less than the pre-defined non-inferiority limit of 10 bpm, non-inferiority of BAT/FF 300/100 to placebo was demonstrated for the primary endpoint. Subjects in the placebo group had a slightly higher WM HR at all visits compared with those in the BAT/FF 300/100 group and there was a slight increase in WM HR in both groups on Day 28 (Fig. 2). On Day 42, WM HR remained stable compared with baseline in the placebo group and had reduced slightly in the BAT/FF 300/100 group (Fig. 2).

**Fig. 3:**
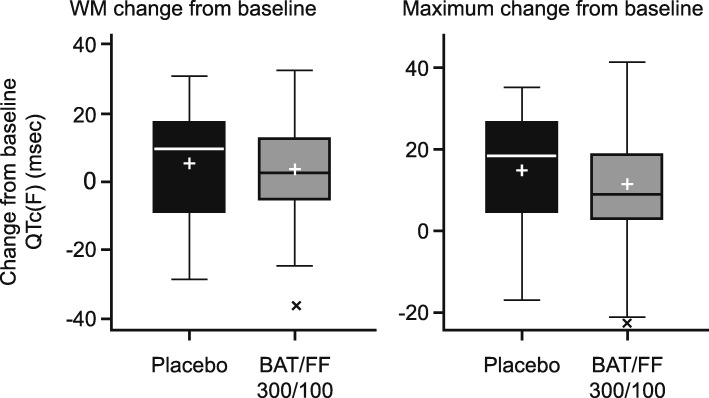
WM and maximum QTc(F) change from baseline to Day 42 (MMRM analyses). BAT/FF 300/100, batefenterol/fluticasone furoate 300/100 μg; QTc(F), QT interval corrected by Fridericia’s method; MMRM, mixed models repeated measures; WM, weighted mean

There were no clinically relevant differences in serial 0–4-h QTc(F) between the treatment groups. The differences between the LS means for the BAT/FF 300/100 and placebo groups ranged from − 5.4 msec (95% CI: − 14.0, 3.2; recorded at 30 min on Day 28) to 7.2 msec (95% CI: − 2.3, 16.7, recorded at 4 h on Day 1). There were also no clinically relevant differences between the treatment groups in the WM QTc(F) and maximum QTc(F) (Fig. 3), both of which remained stable in both treatment groups on Days 1, 28, and 42. Overall, there were no effects on mean HR at Day 42, measured using Holter monitoring in subjects with at least 16 of 24 h of analyzable data (Additional file 1: Table S3). There were no clinically relevant changes in vital signs, pulse rate (Additional file 1: Table S3) or systolic and diastolic BP in either treatment group.

Mean blood eosinophil counts are provided in Table S4. No subject had a mean eosinophil count above 0.3 × 10^9^ cells/L at any time point.

A summary of treatment-emergent AEs is presented in Table 2 and Table S5. Similar proportions of subjects in the BAT/FF 300/100 and placebo groups had AEs. Two subjects in the BAT/FF 300/100 group had AEs that led to permanent discontinuation of study treatment or withdrawal from the study (Table 2), including one subject with a serious AE of severe pneumonia that occurred post treatment and was not considered drug-related, and one with non-serious AEs of moderate vomiting and severe gastroenteritis that was also not considered related to study drug. No deaths were reported.

**Table 2:** Summary of treatment-emergent AEs

n, %	BAT/FF 300/100 (***n*** = 42)	Placebo (***n*** = 20)
Any AE	16 (38)	7 (35)
Drug-related AE	6 (14)	0
AE leading to discontinuation	2 (5)	0
Serious AE	1 (2)^a^	0
Fatal AE	0	0
AEs reported in ≥2 subjects in any treatment group by preferred term^b^		
Dysgeusia	4 (10)	0
Nasopharyngitis	3 (7)	1 (5)
Diarrhea	3 (7)	0
Cough	2 (5)	0

*AE* adverse event, *BAT/FF 300/100* batefenterol/fluticasone furoate 300/100 μg

^a^The single serious AE developed post treatment. ^b^See Table S5 for all AEs experienced during the study

The most common AEs in subjects in the BAT/FF 300/100 group were dysgeusia, nasopharyngitis, diarrhea, and cough (Table 2), all of which were mild in intensity, except for nasopharyngitis, which was mild-to-moderate in intensity. AEs considered possibly related to BAT/FF 300/100 included dysgeusia (altered taste perception) and cough in all patients who reported them. The rates of COPD exacerbations were low, with one case each in the BAT/FF 300/100 group (on treatment) and placebo group (post treatment). The incidence of pneumonia was also low, with 2 cases in the BAT/FF 300/100 group, one of which occurred post treatment, and the other that led to study withdrawal, neither of which were considered possibly related to study treatment.

### Efficacy

**Fig. 4:**
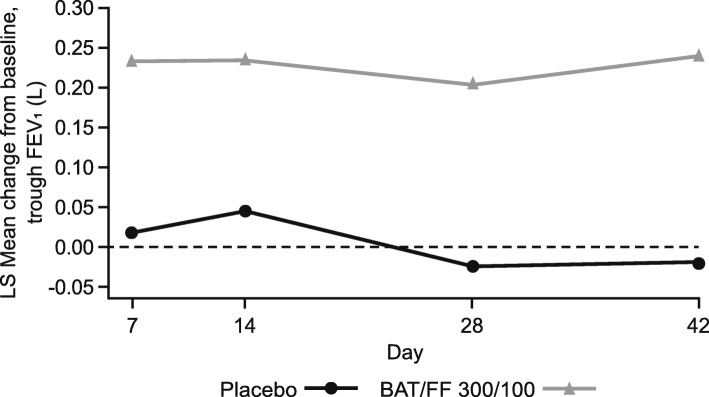
LS mean change from baseline in trough FEV_1_. BAT/FF 300/100, batefenterol/fluticasone furoate 300/100 μg; FEV_1_, forced expiratory volume in 1 s; LS, least squares

Treatment differences in favor of BAT/FF 300/100 over placebo were observed for the spirometry exploratory efficacy endpoint. LS mean changes from baseline in FEV_1_ were consistently higher in the BAT/FF 300/100 group compared with the placebo group, with the largest difference observed on Day 42 (0.262 L, 95% CI: 0.165, 0.359) (Fig. 4, Table 3). No clinically relevant mean difference from placebo in rescue albuterol medication use during a 24-h period (averaged over Weeks 1 to 6) was observed with BAT/FF 300/100 (− 0.17 occasions per day, 95% CI: − 0.75, 0.42) (Table 3). LS mean differences in CAT score (− 1.747, 95% CI: − 4.563, 1.042) and SGRQ-C score (− 1.647, 95% CI: − 8.133, 4.839) between BAT/FF 300/100 and placebo at Day 42 indicated a tendency for a larger improvement with BAT/FF 300/100 (Table 3).

**Table 3:** Efficacy results

	BAT/FF 300/100 (***n*** = 42)	Placebo (***n*** = 20)
Trough FEV_1_ at Day 42, L^a^	*n* = 42	*n* = 20
LS mean change (SE)	0.24 (0.03)	−0.02 (0.04)
Difference vs. placebo (95% CI)	0.26 (0.17, 0.36)	–
Rescue medication use Weeks 1–6, number of occasions/day^b^	*n* = 37	*n* = 16
LS mean change (SE)	−0.50 (0.15)	−0.33 (0.24)
Difference vs. placebo (95% CI)	−0.17 (− 0.75, 0.42)	–
CAT score at Day 42^b^	*n* = 35	*n* = 18
LS mean change (SE)	−1.86 (0.78)	−0.11 (1.14)
Difference vs. placebo (95% CI)	−1.75 (−4.54, 1.04)	–
SGRQ score at Day 42^b^	*n* = 34	*n* = 18
LS mean change (SE)	−3.13 (1.79)	−1.48 (2.62)
Difference vs. placebo (95% CI)	−1.65 (− 8.13, 4.84)	–

^a^MMRM analysis; ^b^ANCOVA model

*ANCOVA* analysis of covariance, *BAT/FF 300/100* batefenterol/fluticasone furoate 300/100 μg, *CAT* COPD Assessment Test, *CI* confidence interval, *FEV*_*1*_ forced expiratory volume in 1 s, *LS* least squares, *MMRM* mixed models repeated measures, *SE* standard error, *SGRQ* St George’s Respiratory Questionnaire

### Pharmacokinetics-pharmacodynamics

Systemic exposure to BAT 300 μg was low; geometric mean maximum plasma concentration was 82.4 ng/L (95% CI: 67.4, 100.7) on Day 42. This low systemic exposure to BAT correlated with the lack of a clinically relevant effect on the 0–4-h WM or maximum HR, 0–4-h WM or maximum QTc(F) and 0–4-h WM or maximum fasting glucose levels, and 0–4-h WM or minimum fasting potassium levels (Table 4).

**Table 4:** LS mean changes from baseline in fasting glucose and potassium concentrations, Day 42 (ANCOVA model)

	BAT/FF 300/100 (***n*** = 42)^**a**^	Placebo (***n*** = 20)
0–4-h fasting glucose concentration on Day 42		
WM mean, mmol/L	*n* = 30	*n* = 20
LS mean change (SE)	−0.146 (0.200)	0.026 (0.263)
Difference vs. placebo (95% CI)	−0.173 (− 0.839, 0.493)	–
Maximum	*n* = 31	*n* = 20
LS mean change (SE)	0.434 (0.272)	0.279 (0.363)
Difference vs. placebo (95% CI)	0.154 (−0.756, 1.065)	–
0–4-h fasting potassium concentration on Day 42		
WM, mmol/L	*n* = 29	*n* = 19
LS mean change (SE)	−0.107 (0.042)	−0.046 (0.055)
Difference vs. placebo (95% CI)	−0.062 (− 0.200, 0.077)	–
Minimum	*n* = 29	*n* = 19
LS mean change (SE)	−0.279 (0.045)	−0.199 (0.060)
Difference vs. placebo (95% CI)	−0.080 (− 0.229, 0.070)	–

^a^For each endpoint, subjects with insufficient data were excluded from analyses

*ANCOVA* analysis of covariance model, *BAT/FF 300/100* batefenterol/fluticasone furoate 300/100 μg, *CI* confidence interval, *LS* least squares, *h* hour, *SE* standard error, *WM* weighted mean

## Discussion

This study was undertaken to determine the safety and tolerability of BAT/FF 300/100 administered once daily via a DPI for 6 weeks in subjects with stable COPD, compared with placebo, with a particular focus on CV safety.

This study was powered to show non-inferiority between BAT/FF 300/100 and placebo for the primary endpoint of change in 0–4-h WM HR. ECG findings showed small and similar changes from baseline in QTc(F) intervals between BAT/FF 300/100 and placebo and changes in HR based on ECG and 24-h Holter monitoring were also small. No new clinically relevant safety signals were identified during 6 weeks of treatment with BAT/FF 300/100, including no clinically relevant effects on vital signs, ECGs, blood glucose, and potassium levels.

These safety and tolerability findings are in line with earlier studies of BAT [14, 15]. In a PD study, BAT had no significant effect on maximum HR, glucose, or QTc(F) 0–4 h compared with placebo after 14 days of treatment in patients with COPD, and was associated with a small decrease in potassium 0–4 h [15]. In a Phase IIb study, 28 days’ treatment with BAT administered via DISKUS had no effect on glucose, potassium, HR, and BP and a limited effect on QTc(F) in patients with moderate to severe COPD [14].

It is conceivable that subjects randomized to placebo could have experienced more dyspnea, which could have adversely impacted the cardiovascular system (e.g. increased heart rate) and minimized detection of the effect of the investigational product on cardiovascular safety. However, only one subject (placebo group) reported dyspnea in this trial, which makes it unlikely this affected the overall cardiovascular safety data.

The ECG findings for BAT/FF in this study are in line with previous CV safety data with LAMAs and LABAs for the treatment of COPD. A recent meta-analysis demonstrated that LABAs do not increase the risk of fatal CV events in patients with COPD, even in long-term trials and in patients with severe COPD [16]. A post hoc analysis of data from the randomized, double-blind, placebo-controlled Towards a Revolution in COPD Health (TORCH) trial demonstrated that LABAs alone, ICS alone, or the combination given for 3 years reduced adverse CV events in patients with moderate to severe COPD compared with placebo [17]. It could be argued that participants in clinical trials were likely to be at lower risk of CV events than ‘real-life’ individuals with COPD; however, a study in patients with moderate COPD and high CV risk demonstrated that treatment with LABAs alone, ICS alone, or the combination did not adversely affect CV outcomes in these patients [18]. The CV safety of LAMAs was assessed in the TIOtropium Safety and Performance In Respimat® (TIOSPIR) trial and the 4-year Understanding Potential Long-term Impacts on Function with Tiotropium (UPLIFT) trial, and was confirmed in a post hoc analysis in subjects in the UPLIFT trial who experienced cardiac effects for which they would have been excluded at study baseline [19]. In addition, dual bronchodilation with LAMA/LABA did not increase CV risk compared with the individual mono-components in a meta-analysis of data from 23,168 patients with COPD [20].

Altered taste perception (dysgeusia) was the most commonly reported AE in this study. These events were considered possibly related to BAT/FF 300/100 and are consistent with those reported in earlier studies with BAT [14]. The AEs of nasopharyngitis, cough, and diarrhea were also noted in previous clinical trials with BAT. In a 28-day dose-ranging study of BAT given once daily (100, 400, and 800 μg) and twice daily (100, 200, and 400 μg) via DISKUS, the most common AEs reported in ≥3% in any group were headache, cough, dysgeusia, and nasopharyngitis [14].

There were differences in the LS mean changes from baseline in trough FEV_1_ in favor of BAT/FF 300/100 versus placebo, with the largest difference observed on Day 42. Although this study was not designed to robustly assess the efficacy of BAT/FF 300/100, its effects on trough FEV_1_ are consistent with those of approved dual bronchodilators (20). However, there were no clinically relevant LS mean treatment differences in the use of rescue albuterol between treatment arms.

Due to the exclusion criteria of the study, all subjects participating in this trial had stable COPD with no moderate or severe exacerbations 1 year prior to screening. In addition, a number of COPD medications were not permitted prior to screening. As such, the results may not be generalizable to a wider COPD population, and safety in patients with unstable COPD has not been investigated.

## Conclusions

The safety and tolerability of BAT/FF 300/100 in patients with COPD was demonstrated in this study. BAT/FF 300/100 was non-inferior to placebo for change from baseline in 0–4-h WM HR (measured using ECG) on Day 42, had no clinically relevant effect on QTc(F), HR measured using Holter monitoring, pulse rate, BP, and fasting glucose and potassium concentrations compared with placebo. BAT/FF 300/100 was well tolerated, with similar proportions of patients reporting AEs as in the placebo group and AEs consistent with previous clinical trials of BAT. AEs were consistent with the pharmacology of BAT/FF and were as expected in a COPD population, and with the exception of dysgeusia, no new safety signals were identified; however, the reports of dysgeusia did not lead to study participant discontinuation. In addition, exploratory efficacy analysis suggested treatment differences in favor of BAT/FF 300/100 over placebo, most notably a significant greater increase in trough FEV_1_ at Day 42. Future studies should address not only long-term safety (e.g. 1 year) of BAT/FF 300/100 in patients with COPD, but also the impact this regimen would have on reducing COPD exacerbation rates.

## Supplementary information

**Additional file 1: Supplementary Appendix.** Study inclusion criteria and exclusion criteria. **Table S1.** Medications not permitted prior to Screening (Visit 1; time interval specified) and throughout the study. **Table S2.** Analysis of 0–4-h WM HR at Days 1, 28, and 42 using MMRM (bpm). **Table S3.** Analysis of Holter findings and pulse rate. **Table S4.** Eosinophil count over time. **Table S5.** Adverse events experienced by ≥1 subject.

## Data Availability

The datasets generated and/or analysed during the current study are available in the US National Library of Medicine Clinical Trials repository, https://clinicaltrials.gov/ct2/show/results/NCT02573870.

## Abbreviations

AE: Adverse event
ANCOVA: Analysis of covariance
BAT: Batefenterol
BMI: Body mass index
BP: Blood pressure
bpm: Beats per minute
CAT: COPD Assessment Test
CI: Confidence interval
COPD: Chronic obstructive pulmonary disease
CV: Cardiovascular
DPI: Dry powder inhaler
ECG: Electrocardiogram
FEV_1_: Forced expiratory volume in 1 s
FF: Fluticasone furoate
GCP: Good Clinical Practice
HR: Heart rate
ICS: Inhaled corticosteroid
ITT: Intent-to-treat
LABA: Long-acting β_2_-adrenergic agonists
LAMA: Long-acting muscarinic antagonists
LS: Least squares
MABA: Muscarinic antagonist, β_2_ agonist
MMRM: Mixed model repeated measures
PD: Pharmacodynamics
PK: Pharmacokinetics
PRO: Patient-reported outcome
PRN: Pro re nata (when necessary)
QD: Once daily
QTc(F): Fridericia’s corrected QT interval
SD: Standard deviation
SE: Standard error
SGRQ: St George’s Respiratory Questionnaire
US: United States
V: Visit
VI: Vilanterol
WM: Weighted mean
