# Supplementary material for: A randomized, controlled, repeat-dose study of batefenterol/fluticasone furoate compared with placebo in the treatment of COPD
Source: BMC Pulm Med. 2020 May 4;20:119. doi: 10.1186/s12890-020-1153-7 (PMC7199364; doi:10.1186/s12890-020-1153-7)
Supplement: Supplementary file 1 — Additional file 1: Supplementary Appendix. Study inclusion criteria and exclusion criteria. Table S1. Medications not permitted prior to Screening (Visit 1; time interval specified) and throughout the study. Table S2. Analysis of 0–4-h WM HR at Days 1, 28, and 42 using MMRM (bpm). Table S3. Analysis of Holter findings and pulse rate. Table S4. Eosinophil count over time. Table S5. Adverse events experienced by ≥1 subject. [file 12890_2020_1153_MOESM1_ESM.docx]

**Supplementary Appendix**

**Study inclusion criteria**

A subject was eligible for inclusion in this study if all of the following criteria were met:

1. Type of subject: Outpatient.
2. Informed consent: Capable of giving signed informed consent, which included

being capable of compliance with the requirements and restrictions listed in the

consent form and in the protocol.

1. Age and sex: Male and female subjects, ≥40 years of age at the time of

signing the informed consent.

Female subjects were eligible if they were not pregnant (confirmed by a urine human chorionic gonadotrophin test), not lactating, and at least one of the following conditions applied:

a. Non-reproductive potential defined as:

- Pre-menopausal females with one of the following:
  - Documented tubal ligation
  - Documented hysteroscopic tubal occlusion procedure with follow-up

confirmation of bilateral tubal occlusion

- - Hysterectomy
  - Documented bilateral oophorectomy
- Post-menopausal defined as 12 months of spontaneous amenorrhea. Females taking hormone replacement therapy (HRT) and whose menopausal status was in doubt were required to use one of the highly effective contraception methods if they wished to continue their HRT during the study. Otherwise, they were required to discontinue HRT to allow confirmation of post-menopausal status prior to study enrollment.

b. Reproductive potential and agreed to follow one of the options listed below 30 days prior to the first dose of study medication and until at least 5 terminal half-lives OR until any continuing pharmacologic effect had ended, whichever was longer after the last dose of study medication and completion of the follow-up visit.

- Contraceptive subdermal implant that met the standard operating procedure (SOP) effectiveness criteria (ie, have a failure rate of <1% per year when used consistently and correctly and, when applicable, in accordance with the product label).
- Intrauterine device or intrauterine system that met the SOP effectiveness criteria (ie, a failure rate of<1% per year when used consistently and correctly and, when applicable, in accordance with the product label)
- Oral contraceptive, either combined or progestogen alone
- Injectable progestogen
- Contraceptive vaginal ring
- Percutaneous contraceptive patches
- Male partner sterilization with documentation of azoospermia prior to the

female subject’s entry into the study, with the male being the sole partner of the female subject

These permitted methods of contraception are only effective when used consistently,

correctly, and in accordance with the product label. The investigator was responsible for ensuring that subjects understood how to use these methods of contraception properly.

1. Chronic obstructive pulmonary disease (COPD) diagnosis: An established clinical history of COPD in accordance with the definition by the American Thoracic Society (ATS)/European Respiratory Society (ERS) (1) as follows:

*“Chronic obstructive pulmonary disease is a preventable and treatable disease state*

*characterized by airflow limitation that is not fully reversible. The airflow limitation is*

*usually progressive and is associated with an abnormal inflammatory response of the*

*lungs to noxious particles or gases, primarily caused by cigarette smoking.”*

1. COPD disease severity: A post-albuterol forced expiratory volume in 1 second(FEV_1_)/forced vital capacity ratio of ≤0.70 and a post-albuterol FEV_1_ ≥30 and ≤80% of predicted normal values calculated using the ERS Global Lung Function Initiative reference equations at Visit 1 (2).
2. Smoking history: Current or former cigarette smokers with a history of cigarette smoking of ≥10 pack-years at Visit 1. Former smokers were defined as those who had stopped smoking for at least 6 months prior to Visit 1.

Number of pack-years = (number of cigarettes per day / 20) x number of years smoked

(eg, 20 cigarettes per day for 10 years, or 10 cigarettes per day for 20 years both equal

10 pack-years).

Note: Pipe and cigar use cannot be used to calculate pack-year history.

**Study exclusion criteria**

A subject was not eligible for inclusion in this study if any of the following criteria were met:

1. Asthma: Had a current diagnosis of asthma (subjects with a prior history of asthma were eligible if they had a current diagnosis of COPD).
2. Other respiratory disorders: Known α-1 antitrypsin deficiency, active lung infections (such as tuberculosis), and lung cancer were absolute exclusionary conditions. Other excluded conditions included and were not limited to clinically significant bronchiectasis, pulmonary hypertension unrelated to COPD, sarcoidosis, or interstitial lung disease. Or a subject who, in the opinion of the investigator, had any other significant respiratory conditions in addition to COPD.
3. Other diseases/abnormalities: Subjects with historical or current evidence of clinically significant cardiovascular, neurological, psychiatric, renal, hepatic, immunological, endocrine (including uncontrolled diabetes or thyroid disease), or hematological abnormalities that were uncontrolled and/or a previous history of cancer in remission for <5 years prior to Visit 1 (except localized carcinoma of the skin that had been resected for cure).

‘Significant’ was defined as any disease that, in the opinion of the investigator, would have put the safety of the subject at risk through participation, or which would have affected the efficacy or safety analysis if the disease/condition had exacerbated during the study.

1. Poorly controlled COPD: Defined as the occurrence of ‘acute worsening of COPD that is managed with corticosteroid and/or antibiotics or that requires treatment prescribed by a physician in the 6 weeks prior to Screening (Visit 1)’, or ‘subjects who are hospitalized due to acute worsening of COPD within 12 weeks of Visit 1’.
2. History of COPD exacerbation: Subject who had had more than one exacerbation (moderate or severe) within the 12 months prior to Visit 1.
3. Pneumonia and lower respiratory tract infection: Subjects with lower respiratory tract infection that had required the use of antibiotics within 6 weeks prior to Visit 1; or subjects hospitalized due to pneumonia within 12 weeks of Visit 1.
4. Use of long-term oxygen therapy: Oxygen therapy prescribed for >12 h a day. As-needed oxygen use (ie, ≤12 h per day) was not exclusionary.
5. Nebulized therapy: Regular use (prescribed for use every day, not for as-needed

use) of short-acting bronchodilators (eg, albuterol) via nebulized therapy.

1. Lung resection: Lung volume reduction surgery within the 12 months prior to Visit 1.
2. Clinically significant abnormal laboratory finding at Visit 1.
3. Liver disease: Current or chronic history of liver disease, or known hepatic or biliary abnormalities (except Gilbert's syndrome or asymptomatic gallstones).
4. Presence of hepatitis B surface antigen, or positive hepatitis C antibody test result at Screening. Subjects with positive hepatitis C antibody due to prior resolved disease could be enrolled only if a confirmatory negative hepatitis C RNA polymerase chain reaction test was obtained.
5. Abnormal and clinically significant findings from 12-lead electrocardiogram (ECG) performed at Visit 1. Site investigators were provided with ECG overread conducted by a centralized independent cardiologist, to assist in evaluation of subject eligibility. For this study, an abnormal and clinically significant ECG that precluded a subject from entering the trial was defined as a
   12-lead tracing that was interpreted as, but not limited to, any of the following:

- Sinus bradycardia <45 beats per minute (bpm) confirmed by 2 additional readings at least
  5 min apart.
- Sinus tachycardia ≥110 bpm confirmed by 2 additional readings at least 5 min apart.
- Multifocal atrial tachycardia (wandering atrial pacemaker with rate >100 bpm)
- PR interval >240 msec
- Evidence of Mobitz II second-degree or third-degree atrioventricular block
- Pathological Q waves (defined as wide [>0.04 seconds] and deep [>0.4 mV (4 mm

with 10 mm/mV setting)] or >25% of the height of the corresponding R wave,

provided the R wave was >0.5 mV [5 mm with 10 mm/mV setting]), appearing in

at least 2 contiguous leads.
Note: Prior evidence of pathological Q waves (ie, ECG obtained at least 12 months prior) that were unchanged was not exclusionary; and the investigator determined if the subject was precluded from entering the study.

- Evidence of ventricular ectopic couplets, bigeminy, trigeminy, or multifocal

premature ventricular complexes.

- For subjects without complete right bundle branch block: QT corrected (Fridericia's formula) (QTc[F]) ≥450 msec or an ECG that was unsuitable for QT measurements (eg, poor defined termination of the T wave).
- For subjects with complete right bundle branch block: QTc(F) ≥480 msec or an ECG that was unsuitable for QT measurements (eg, poor defined termination of the T wave).

Note: All potentially exclusionary QT measurements should have been confirmed by
2 additional readings at least 5 min apart.

- ST-T wave abnormalities (excluding non-specific ST-T wave abnormalities)

Note: Prior evidence (ie, ECG obtained at least 12 months prior) of ST-T waves that were unchanged were not exclusionary and the investigator determined if the subject was precluded from entering the study.

- Clinically significant conduction abnormalities (eg, Wolff-Parkinson-White syndrome or bifascicular block defined as complete left bundle branch block or complete right bundle branch block with concomitant left fascicular block).
- Clinically significant arrhythmias (eg, atrial fibrillation with rapid ventricular

response, ventricular tachycardia).

1. Medication prior to spirometry: Unable to withhold albuterol for the 4-h period required prior to spirometry testing at each study visit.
2. Excluded medications: Use of the medications listed in Table S1 was not permitted within the defined time intervals prior to Visit 1and throughout the study.
3. Drug or alcohol abuse: A known or suspected history of alcohol or drug abuse within 2 years prior to Visit 1.
4. Contraindications: Any history of allergy or hypersensitivity to any anticholinergic/muscarinic receptor antagonist, β_2_-agonist, sympathomimetic, corticosteroid (intranasal, inhaled or systemic), lactose/milk protein, or a medical condition such as narrow-angle glaucoma, prostatic hypertrophy or bladder neck obstruction, that, in the opinion of the study physician contraindicated study participation or use of an inhaled long-acting muscarinic antagonist (LAMA), long-acting β-agonist (LABA) or inhaled corticosteroid.
5. The subject had participated in a clinical trial and had received an investigational product within the following time period prior to the first dosing day in the current study: 30 days, 5 half-lives or twice the duration of the biological effect of the investigational product (whichever was longer).
6. Affiliation with investigator site: A subject was not eligible for this study if he/she was an immediate family member of the participating investigator, sub-investigator, study coordinator, or employee of the participating investigator.
7. Inability to read: In the opinion of the investigator, any subject who was unable to read and/or would not have been able to complete a diary.
8. Questionable validity of consent: Subjects with a history of psychiatric disease, intellectual deficiency, poor motivation or other conditions that limited the validity of informed consent to participate in the study.
9. Non-compliance: Subjects at risk of non-compliance, or unable to comply with the study procedures. Any infirmity, disability, or geographic location that would have limited compliance for scheduled visits.

**Table S1.** Medications not permitted prior to Screening (Visit 1; time interval specified) and throughout the study

| **Medication** | **Time interval** |
| --- | --- |
| Depot corticosteroids | 12 weeks |
| Systemic, oral, or parenteral corticosteroids | 6 weeks |
| Antibiotics (for lower respiratory tract infection) | 6 weeks |
| Cytochrome P450 3A4 strong inhibitors and P-glycoprotein  inhibitors including but not limited to antiretrovirals (protease  inhibitors) (eg, indinavir, nelfinavir, ritonavir, saquinavir,  atazanavir); imidazole and triazole anti-fungals (eg,  ketaconazole, itraconazole, voriconazole); clarithromycin,  telithromycin, troleandomycin, mibefradil, cyclosporin,  nefazodone | 4 weeks |
| LABA/ICS combination products | 4 weeks |
| ICS | 4 weeks |
| Phosphodiesterase 4 (PDE4) inhibitors (roflumilast) | 1 week |
| LABA/LAMA combination (eg, vilanterol /umeclidinium bromide) | 1 week |
| Once-daily β_2_-agonist (eg, olodaterol and Indacaterol) | 1 week |
| LAMAs (tiotropium, aclidinium, glycopyrronium, umeclidinium) | 1 week |
| Theophylline preparations | 48 hours |
| Oral leukotriene inhibitors (zafirlukast, montelukast, zileuton | 48 hours |
| Oral β_2_-agonists  Long acting  Short acting | 48 hours  12 hours |
| Inhaled LABA (eg salmeterol, formoterol) | 48 hours |
| Inhaled sodium cromoglycate or nedocromil sodium | 24 hours |
| Inhaled short-acting β_2_-agonists* | 4 hours |
| Inhaled short-acting anticholinergics | 4 hours |
| Inhaled short-acting anticholinergic/short-acting β_2_-agonist  combination products | 4 hours |

ICS, inhaled corticosteroid; LABA, long-acting β-agonist; LAMA, long-acting muscarinic antagonist.

**Results**

**Table S2.** Analysis of 0–4-h WM HR at Days 1, 28, and 42 using MMRM (bpm)

|  | **BAT/FF 300/100**  **(n=42)** | **Placebo**  **(n=20)** |
| --- | --- | --- |
| Day 1 |  |  |
| LS mean change (SE), bpm | -1.0 (0.7) | 0.3 (1.1) |
| Difference vs. placebo (95% CI) | -1.3 (-4.0, 1.4) | - |
| Day 28 |  |  |
| LS mean change (SE) | 2.5 (1.2) | 3.5 (1.6) |
| Difference vs. placebo (95% CI) | -1.0 (-5.2, 3.1) | - |
| Day 42 |  |  |
| LS mean change (SE) | -1.6 (1.1) | 0.7 (1.5) |
| Difference vs. placebo (95% CI) | -2.2 (-6.2, 1.7) | - |

BAT/FF 300/100, batefenterol/fluticasone furoate 300/100 μg; bpm, beat per minute; CI, confidence interval; HR, heart rate; LS, least squares; MMRM, mixed models repeated measures; SE, standard error; WM, weighted mean.

**Table S3.** Analysis of Holter findings and pulse rate

|  | **BAT/FF**  **300/100**  **(n=42)** | **Placebo**  **(n=20)** |
| --- | --- | --- |
| Mean Holter ECG heart rates (0–24 h) for subjects with at least 16 h of recorded time | | |
| Screening |  |  |
| n | 42 | 19 |
| Mean (SD), bpm | 79.2 (12.22) | 84.2 (8.49) |
| Day 42 |  |  |
| n | 34 | 19 |
| Mean (SD), bpm | 76.3 (11.38) | 84.8 (9.87) |
| Holter ECG findings at Screening and Day 42 | | |
| Screening |  |  |
| n | 42 | 20 |
| Normal, n (%) | 28 (67) | 15 (75) |
| Abnormal, n (%) | 13 (31) | 5 (25) |
| Unable to evaluate, n (%) | 1 (2) | 0 |
| Day 42 |  |  |
| n | 34 | 19 |
| Normal, n (%) | 27 (79) | 12 (63) |
| Abnormal, n (%) | 7 (21) | 7 (37) |
| Unable to evaluate, n (%) | 0 | 0 |
| Analysis of 0–4-h WM pulse rate at Days 1 and 42* | | |
| Day 1 |  |  |
| n | 42 | 20 |
| LS mean change (SE), bpm | -1.48 (0.71) | -0.48 (1.05) |
| Difference placebo (95% CI), bpm | -1.0 (-3.6, 1.6) | - |
| Day 42 |  |  |
| n | 42 | 20 |
| LS mean change (SE), bpm | -2.75 (1.14) | 0.34 (1.54) |
| Difference placebo (95% CI), bpm | -3.1 (-7.0, 0.8) | - |

*MMRM analysis.
BAT/FF 300/100, batefenterol/fluticasone furoate 300/100 μg; bpm, beat per minute; CI, confidence interval; ECG, electrocardiogram; LS, least squares; MMRM, mixed models repeated measures; SD, standard deviation; SE, standard error; WM, weighted mean.

**Table S4.** Eosinophil count over time.

| Mean (SD) eosinophils, 10^9^ cells/L | **BAT/FF 300/100**  **(n=42)** | **Placebo**  **(n=20)** |
| --- | --- | --- |
| Screening | 0.139 (0.0898) | 0.192 (0.1784) |
| Day 1 | 0.148 (0.0895) | 0.196 (0.2244) |
| Day 14 | 0.161 (0.0920) | 0.189 (0.1868) |
| Day 28 | 0.164 (0.0912) | 0.204 (0.1791) |
| Day 42 | 0.152 (0.1068) | 0.173 (0.1625) |
| Early withdrawal | 0.158 (0.893) | - |

BAT/FF 300/100, batefenterol/fluticasone furoate 300/100 μg; SD, standard deviation.

**Table S5.** Adverse events experienced by ≥1 subject

| n, % | **BAT/FF 300/100**  **(n=42)** | **Placebo**  **(n=20)** |
| --- | --- | --- |
| Any adverse event | 16 (38) | 7 (35) |
| Dysgeusia | 4 (10) | 0 |
| Nasopharyngitis | 3 (7) | 1 (5) |
| Diarrhea | 3 (7) | 0 |
| Cough | 2 (5) | 0 |
| Sinusitis | 1 (2) | 1 (5) |
| Arthritis | 1 (2) | 0 |
| Back pain | 0 | 1 (5) |
| Blood creatinine phosphokinase increased | 1 (2) | 0 |
| Blood potassium increased | 1 (2) | 0 |
| Blood pressure increased | 0 | 1 (5) |
| Candida infection | 0 | 1 (5) |
| Dyspnea | 0 | 1 (5) |
| Dysuria | 1 (2) | 0 |
| Ear infection | 1 (2) | 0 |
| Fatigue | 0 | 1 (5) |
| Gastroenteritis | 1 (2) | 0 |
| Gastro-esophageal reflux disease | 1 (2) | 0 |
| Hyperglycemia | 1 (2) | 0 |
| Hypertension | 1 (2) | 0 |
| Hypoxia | 0 | 1 (5) |
| Nausea | 0 | 1 (5) |
| Oral candidiasis | 1 (2) | 0 |
| Paranasal sinus discomfort | 1 (2) | 0 |
| Paranasal sinus hypersecretion | 1 (2) | 0 |
| Pulmonary mass | 1 (2) | 0 |
| Pyrexia | 1 (2) | 0 |
| Sinus congestion | 0 | 1 (5) |
| Skin cancer | 1 (2) | 0 |
| Somnolence | 1 (2) | 0 |
| Vertigo | 0 | 1 (5) |
| Vomiting | 1 (2) | 0 |

BAT/FF 300/100, batefenterol/fluticasone furoate 300/100 μg.

**References**

1. Celli BR, MacNee W, Force AET. Standards for the diagnosis and treatment of patients with COPD: a summary of the ATS/ERS position paper. Eur Respir J. 2004;23(6):932-46.

2. Quanjer PH, Stanojevic S, Cole TJ, Baur X, Hall GL, Culver BH, et al. Multi-ethnic reference values for spirometry for the 3-95-yr age range: the global lung function 2012 equations. Eur Respir J. 2012;40(6):1324-43.
